# Laparoscopic Cholecystectomy Under Combined Spinal and Epidural Anesthesia in the First Trimester of Pregnancy—Case Report and Literature Review

**DOI:** 10.3390/life14111492

**Published:** 2024-11-16

**Authors:** Gabriel-Petre Gorecki, Andrei Bodor, Zoltan-Janos Kövér, Maria-Mihaela Comănici, Romina-Marina Sima, Anca-Maria Panaitescu, Adrian-Vasile Comănici, Emilia Furdu-Lungut, Ancuta-Alina Constantin, Liana Pleș, Andrei Sebastian Diaconescu, Vasile Lungu

**Affiliations:** 1 Department of Anesthesia and Intensive Care, Faculty of Medicine, “Titu Maiorescu” University, 031593 Bucharest, Romania; gabriel.gorecki@prof.utm.ro; 2 Department of Anesthesia and Intensive Care, CF2 Clinical Hospital, 011464 Bucharest, Romania; 3 1st Department of Cardiovascular Anesthesiology and Intensive Care, Prof. Dr. C. C. Iliescu Emergency Institute for Cardiovascular Diseases, 022328 Bucharest, Romania; andreibodor96@gmail.com; 4 Department of General Surgery, CF2 Clinical Hospital, 011464 Bucharest, Romania; kover_zoltan@yahoo.com (Z.-J.K.); vasilelungu.md@gmail.com (V.L.); 5 Department of Immunology, Faculty of Medicine, “Titu Maiorescu” University, 031593 Bucharest, Romania; mihaela.comanici@prof.utm.ro; 6 Department of Clinical Immunology and Molecular Biology, CF2 Clinical Hospital, 011464 Bucharest, Romania; 7 “Carol Davila” University of Medicine and Pharmacy, 020021 Bucharest, Romania; anca.panaitescu@umfcd.ro (A.-M.P.); ancuta-alina.constantin@umfcd.ro (A.-A.C.); liana.ples@umfcd.ro (L.P.); andrei.diaconescu@umfcd.ro (A.S.D.); 8 Department of Obstetrics and Gynecology, The “Bucur” Maternity, “Saint John” Hospital, 040294 Bucharest, Romania; 9 Department of Obstetrics and Gynecology, Filantropia Clinical Hospital Bucharest, 011171 Bucharest, Romania; 10 Department of Endocrinology, Faculty of Medicine, “Titu Maiorescu” University, 031593 Bucharest, Romania; adrian.comanici@prof.utm.ro; 11 Department of Endocrinology, CF2 Clinical Hospital, 011464 Bucharest, Romania; 12 Department of Neurology, Faculty of Medicine, “Titu Maiorescu” University, 031593 Bucharest, Romania; emilia.furdu@spcf2.ro; 13 Department of Neurology, CF2 Clinical Hospital, 011464 Bucharest, Romania; 14 “Marius Nasta” National Institute of Pneumology, 90 Viilor Street, 050159 Bucharest, Romania; 15 General Surgery Department, Fundeni Clinical Institute, 022328 Bucharest, Romania

**Keywords:** laparoscopy, combined spinal and epidural anesthesia (CSEA), early pregnancy, complication, cholecystectomy

## Abstract

Can combined spinal and epidural anesthesia be the gold standard for laparoscopic surgery for pregnant patients? This case report presents a first trimester pregnant patient who was admitted for obstructive jaundice syndrome (pain in the right hypochondrium, nausea, and vomiting). Initially, because of the risk/benefit ratio of pregnancy, the treatment was medical and the patient was immediately discharged because her clinical condition improved, but she was rapidly readmitted to the surgery department because of worsening symptoms. Emergency surgical intervention (laparoscopic cholecystectomy) under combined spinal and epidural anesthesia (CSEA) was performed to reduce the patient’s risks. Since most analgesics are insufficiently studied in pregnancy, analgesia with ropivacaine 0.2% was used on the epidural catheter. No pathological changes were identified in the fetal Doppler ultrasound preoperatively and postoperatively. Similarly to other studies, our case highlights the necessity for cholecystectomy for acute cholecystitis even if the patient is in the first trimester of pregnancy. If the decision is delayed, the morbidity and mortality for mother and fetus become unjustified. The peculiarity of the present report is the type of anesthesia chosen. We consider that combined spinal and epidural anesthesia may become a possible gold standard suitable for laparoscopy in the first trimester of pregnancy.

## 1. Introduction

This paper presents a case report of an 11-week pregnant woman who successfully underwent laparoscopic cholecystectomy under combined spinal and epidural anesthesia. The pregnant patient represents one of the few situations that can put both the surgeon and the anesthesiologist in difficulty. Both anesthetic and surgical procedures have risks, which are exponentially higher in pregnant women compared to non-pregnant women [1,2,3]. In general, the complications of pregnant and non-pregnant patients are the same, but possibly due to the surgical stress and its consequences, we also found additional risks such as spontaneous abortion in the first trimester or premature birth later [1,2,3]. From an anesthetic point of view, safety has not been proven regardless of the trimester of pregnancy, and even more, studies on laboratory animals have demonstrated disorders in fetal neural development [4,5]. However, not all pregnant women present medical problems that can be postponed until the second trimester, which is the optimal gestational period accepted by most studies as optimal for surgical interventions during pregnancy [6]. Due to the fact that the moment when a pathology can become complicated in such a way as to harm both the mother and the child can occur at any time, several studies have concluded that the occurrence of complications leads to the need for surgical intervention regardless of the trimester of pregnancy due to the increased risk of maternal mortality [7]. Cholecystitis during pregnancy is one of the pathologies worthy of consideration [8,9,10]. Gallstones cause inflammation of the gallbladder and are most often the result of changes in hormonal secretion associated with pregnancy, favoring a slow emptying of the gallbladder and the formation of small stones, most often from cholesterol [8]. There is evidence that if cholecystectomy is delayed during pregnancy, the risk of morbidity and mortality increases for both the mother and fetus [8,11,12,13,14]. Acute cholecystitis during pregnancy occurs in any trimester of pregnancy, with almost 40% in the first trimester. There is no guidelines to recommend cholecystectomy across trimesters, and evidence about its management is lacking [14].

With this case report, we support the previously mentioned hypothesis because the pregnant woman was initially treated conservatively (medication to relieve symptoms) and complications (pancreatic inflammation and acute cholecystitis) soon appeared, which increased the risk of morbidity and mortality associated with surgical intervention, but this was lower compared to the risk of untreated cholecystitis. Regarding the surgical method, laparoscopy versus laparotomy, there is growing evidence in favor of laparoscopy. It presents multiple advantages, including shortening the length of hospitalization, reducing postoperative pain, and reducing the need for analgesics used, helping to rehabilitate the patient more quickly [14]. The standard method of anesthesia in the case of laparoscopy was considered to be general anesthesia. We notice an increasing number of publications that support regional anesthesia (spinal anesthesia (SA) or combined spinal and epidural anesthesia (CSEA)) and their advantages compared to general anesthesia [15,16,17,18,19]. Continuous analgesia on the epidural catheter can represent the gold standard for postoperative pain in pregnancy. At the moment, through studies, there is a limited range of analgesic drugs proven to be safe in pregnancy [4,5]. By administering an analgesic dose of ropivacaine on the epidural catheter, we reduce the number and risk of intravenous analgesics via transplacental passage. Using epidural catheter and spinal anesthesia, anesthesia can be administered without opioids, which is less harmful [20]. The hemodynamic impact generated by regional anesthesia can be easily controlled with very small doses of vasopressor. General anesthesia uses drugs from several classes, and thus there is a greater risk of both fetal and maternal exposure (anaphylactic shock, malignant hyperthermia, fetal hypoxia, etc.). There are multiple advantages of regional anesthesia compared to general anesthesia highlighted in different studies [21,22]. This case aims to support the data which underline that regional anesthesia is feasible for cases of laparoscopic cholecystectomy associated with the first trimester of pregnancy.

## 2. Case Presentation

The 37-year-old primigravida patient, in the first trimester of pregnancy (11th gestational week), presents to the medical center for symptoms suggestive of obstructive jaundice (pain in the right hypochondrium, nausea, and vomiting). The patient had no significant medical history. She had no previous surgical interventions or declared pathologies.

At the time of presentation, the patient was hemodynamically and respiratory stable, systolic blood pressure/diastolic blood pressure (SBP/DBP) was 130/58 mmHg, heart rate was 78/min, respiratory rate was 16/min, and peripheral oxygen saturation was 98–100% at an inspiratory oxygen fraction of 21%. Ultrasound performed for maternal gallbladder revealed images with a different echogenicity suggestive of gallstones, without the dilation of the intrahepatic and extrahepatic bile ducts. There was also a pregnancy with normal characteristics for the patient’s gestational age. Laboratory investigations revealed leukocytes—10,870 per mm^3^ with an increase in neutrophils by 80.50%, normal amylase, and an increase in direct bilirubin (0.73 mg/dL) with an increase in total bilirubin (1.5 mg/dL). Also, elevations in alkaline phosphatase (202 IU/L), gamma-glutamyl transferase (GGT) (152 IU/L), aspartate aminotransferase (AST) (217 IU/L), alanine aminotransferase (ALT) (490 IU/L), and C-reactive protein (CRP) (10.67 mg/dL) were identified. The electrocardiogram (ECG) examination and the cardiopulmonary radiography were normal. The diagnosis of lithiasis cholecystitis, associated with hepatocytolysis syndrome, was confirmed. The patient was not aware of the gallstones, even if she experienced similar symptoms before pregnancy, but she did not attend any physician for that condition.

In the first phase, a conservative treatment was decided. It consisted of fluid administration associated with antispasmodic, antiemetic, and analgesic drugs that improved the general condition of the patient. Acetaminophen, drotaverine chlorhydrate, and amoxicillin were used as the first-line treatment. According to this medical improvement, the patient was discharged with recommendations. The laboratory investigations before the patient’s discharge revealed a decrease in leukocytes of 8.570 per mm^3^ with the normalization of the reference interval for neutrophils of 75.70%. There were also decreases in both direct (0.35 mg/dL) and total (0.91 mg/dL) bilirubin. The values of alkaline phosphatase (173 IU/L), GGT (132 IU/L), AST (81 IU/L), and ALT (281 IU/L). C reactive protein was 10 mg/dL at that moment.

The choice for a medical approach instead of a surgical one in this case was based on the risk/benefit ratio for the pregnancy. Even if the surgical intervention would have been an option for the patient, the recommendations are for postponing it for the second trimester of pregnancy in order to have a safety profile. Eleven days after being discharged, the patient’s symptoms worsened at home. She presented more intense pain in the right hypochondrium compared with the previous episode, requiring a new admission in the general surgery department for further investigations. Blood tests at the patient’s admission revealed leukocytes 9.290 per mm^3^ and hemoglobin 11 g/dL with a hematocrit of 32.20%. MCV was 82.6 fl and MCH 28.2 pg, the platelet count was 200.00 × 10^3^/μL, neutrophils were 71.90%, lymphocytes were 21.30%, eosinophils were 0.5%, monocytes were 6.10%, basophils were 0.20%, and amylase was 51.1 U/L. C-reactive protein was 10.2 mg/dL. The hepatocytolysis syndrome that improved during the previous hospitalization reappeared, registering increased values of AST (232 IU/L) and ALT (355 IU/L) in association with changes in direct (1.26 mg/dL) and total (1.89 mg/dL) bilirubin. Increases in alkaline phosphatase (246 IU/L) and GGT (223 IU/L) were also recorded compared to the values from the patient’s discharge. The obstetrical ultrasound evaluation revealed intrauterine a single fetus, amniotic fluid in normal quantity, and the trophoblast of a normal appearance inserted on the anterior wall. The fetal heart rate was present at 165/min and a CRL of 57.7 mm was measured, measurements corresponding to a normal-developing pregnancy at week 11–12 of pregnancy. An ultrasound examination of the gallbladder was also performed to reveal any progression or complication of the pathology. The presence of four images with modified echogenicity with a size of 5–7 mm was identified, suggestive of gallstones without dilatation of the intrahepatic bile ducts. The gallbladder walls were thicker at the ultrasound examination.

Fluid and analgesic therapy with antispasmodic and antiemetic drugs was initiated. The patient’s condition progressively worsened during hospitalization, and a prophylactic antibiotic was chosen to prevent a potential infection and the onset of sepsis [23]. The antibiotic was ceftriaxone two grams per day. The reason for its choice was based on several studies which confirm that it is safe during pregnancy [24]. The U.S. Food and Drug Administration (FDA) considers ceftriaxone a pregnancy category B medicine [25]. Also, there is no FDA-approved category A for antibiotics at this moment.

Additional investigations, associated with the patient’s progressively worsening condition, led to the diagnosis of acute cholecystitis with hepatocytolysis syndrome and pancreatic reaction. For this reason, a multidisciplinary team consisting of a general surgeon in collaboration with an obstetrician and an anesthetist analyzed the case in order to find a favorable risk/benefit therapeutic solution. It was concluded that due to the negative evolution of the patient under antibiotic therapy associated with analgesic and fluid resuscitation, it is possible that the cholecystitis is acute and there is a risk of superinfection later if the surgical intervention is not performed. The chosen surgical technique was laparoscopy, and the proposed anesthetic procedure was combined spinal and epidural anesthesia due to the high risks of general anesthesia. An informed consent form was signed by the patient, which included the risks for both surgery and anesthesia, as well as the possibility of conversion from regional to general anesthesia and the subsequent consequences. The patient followed the preoperative preparation according to the protocols.

The patient was admitted to the operating room for surgery. The surgical team was composed of experienced general surgeons. Intraoperative monitoring consisted of electrocardiogram (EKG) measured in two leads (DII and V5) with a registered heart rate between 86 and 76 bpm. No EKG changes were identified during the intervention. The peripheral oxygen saturation (SpO_2_) was monitored with a pulse oximeter. The value recorded throughout the surgery was 100%. Oxygen therapy was used with additional O_2_ on the face mask with a flow rate of 6 L/min to prevent hypoxia and to ensure a satisfactory oxygen reserve. Blood pressure was measured non-invasively (once every 3 min). The baseline blood pressure measurement was SBP/DBP 132/62 mmHg with a mean arterial pressure (MAP) of 85 mmHg. After spinal anesthesia, blood pressure decreased to SBP/DBP 105/50 mmHg (MAP = 68 mmHg), which is why a low-dose vasopressor was used with a subsequent increase in values to SBP/DBP 128/68 mmHg (MAP = 88 mmHg). ETCO_2_ (surrogate for partial pressure of carbon dioxide—PaCO_2_) was measured non-invasively. The ETCO_2_ values recorded were between 33 and 36. An 18 G peripheral venous catheter ensured intraoperative perfusion as well as drug administration during the intervention. The antibiotic prophylaxis used was ceftriaxone in a single dose of two grams. A bolus of 500 mL NaCl 0.9% solution was administered immediately after the anesthetic procedure to ensure perfusion to the organs during CO_2_ insufflation in the peritoneal cavity and to prevent a steep drop in mean blood pressure due to sympatholysis given by epidural anesthesia. The gallbladder was removed through a skin incision made at the insertion site of a trocar. The total amount of fluids used intraoperatively was 1000 mL of NaCl 0.9%, and the duration of the intervention was 40 min.

Premedication: The patient’s anxiolysis and comfort were ensured by midazolam (1 mg) administered during the combined spinal–epidural anesthesia (CSEA).

Anesthesia technique: The position chosen for anesthesia was the sitting position. The area of interest was disinfected with chlorhexidine 2% with the rules of asepsis and antisepsis respected according to the guidelines [26]. Local anesthesia with 1% lidocaine (5 mL) was injected subcutaneously to ensure patient analgesia during the procedure. The chosen intervertebral space was L2–L3. A 27 G pencan needle was used for spinal anesthesia. A total of 12 mg of hyperbaric bupivacaine 0.5% was injected. Later, an epidural catheter was inserted at the same level. The epidural space was identified at 7 cm, and the epidural catheter was fixed to the skin at approximately 12 cm. A bolus of 15 mL of ropivacaine 0.75% was injected into the epidural catheter. The patient was placed in the Trendelenburg position (10 degrees) to ensure the ascension of anesthesia. After the ropivacaine injection, we waited about 20 min for the installation of the sensory and motor block. The sensory block testing was performed with the help of the patient, who was asked to answer some questions about the pain intensity felt at various levels.

After the anesthetic effect was established, a Veress needle was used to insufflate CO_2_ into the abdominal cavity. The laparoscopy was performed following the American technique. The 10 mm optic trocar was subxifoidian, and the other two trocars of 5 mm were on the right side of the abdomen on the medioclavicular right line. The initially chosen intra-abdominal pressure was 12 mmHg. Once this value was reached, the trocars were inserted. During the inspection of the abdominal cavity, the gallbladder was identified, which had an inflamed aspect, dilated in volume with the presence of stones at this level. Shortly after the start of surgery (10–15 min), the patient complained of intense pain in the right shoulder (due to intra-abdominal pressure on the diaphragm and phrenic nerve). The intra-abdominal pressure at that time was 12 mmHg. We asked the surgical team for a reduction from 12 mmHg to 10 mmHg. The pressure of 10 mmHg ensured no problems regarding the surgical act. Also, the patient’s symptoms improved considerably after this change. Due to the fact that the patient remained conscious during the surgical intervention, a high degree of satisfaction was observed in her attitude. The surgical time was 45 min.

**Figure 1:**
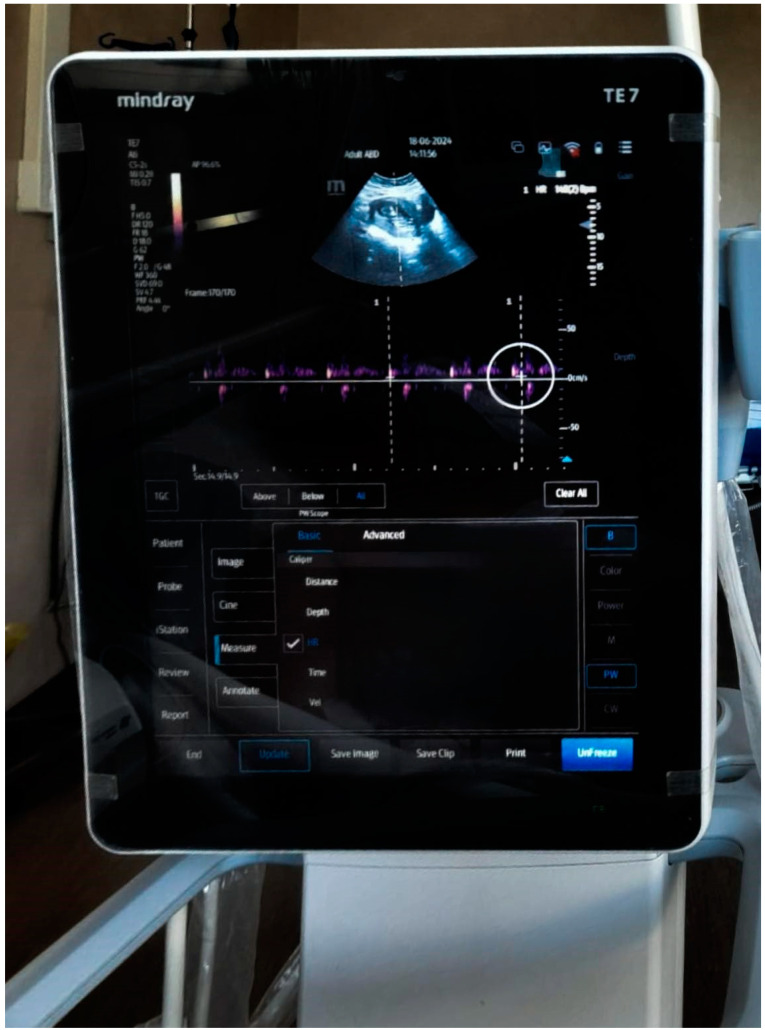
Fetal heartbeat ultrasound performed immediately postoperatively.

The patient was transferred postoperatively to the intensive care unit where she was monitored for 24 h after the intervention. Patient monitoring consisted of EKG, peripheral oxygen saturation, diuresis, and a non-invasive measure of systolic and diastolic blood pressures. The medication consisted of hydration with Ringer’s serum and continuous analgesia on the epidural catheter with ropivacaine 0.2% at a rate of 6–10 mL/h with changes made depending on symptoms. The surgical intervention was uneventful. There were no gallstones or gallbladder spillage in the peritoneal cavity. The gallbladder was intactly removed in an EndoBag from the peritoneal cavity. This fact imposed no drainage of the peritoneal cavity. The gallbladder was sent to the pathologist, but the bile had no bacteriological examination (no standard procedure in our hospital). Additional analgesia was achieved with one gram of paracetamol every eight hours. The antibiotic therapy started preoperatively with two grams of ceftriaxone and was continued both intraoperatively and postoperatively. Antithrombotic prophylaxis was performed with enoxaparin and stress ulcer prophylaxis with pantoprazole. Doppler ultrasound of the fetal heartbeat was performed immediately postoperatively and when the patient was discharged from the intensive care unit. The examination revealed no changes compared to the preoperative examination (Figure 1). The patient was discharged from the hospital 24 h after surgery. She was monitored for 24 h in the intensive care unit and after the surgical and obstetrical evaluation, she was discharged from hospital.

The pathological exam confirmed acute cholecystitis. The patient received Enoxaparin 0.4 mL/day for the following 5 days after the surgical intervention. The follow-up was performed 7 days after the surgical intervention. The patient has no medical complains, and the obstetrical ultrasound revealed normal characteristics of the pregnancy. The pregnancy presented no complication, and the patient gave birth by cesarean section due to obstetrical reasons at 39 weeks of pregnancy to a healthy baby girl. The follow-up after the cesarean section was uneventful, with no consequence caused by the laparoscopy from pregnancy.

## 3. Discussion

The present paper presents the case of a 37-year-old pregnant woman who successfully underwent laparoscopic cholecystectomy under the effect of combined spinal and epidural anesthesia (CSEA). Pregnant patients who develop cholecystitis are at additional risk of generalized peritonitis and subsequent sepsis [14]. These complications can lead to miscarriage, premature birth, and stillbirth [14]. Evidence-based medicine shows that laparoscopic cholecystectomy has no additional risk for mother and fetus compared to open surgery [14]. If there is the necessary equipment and qualified physicians, laparoscopy may be considered an equivalent approach to open surgery [14]. Laparoscopic surgical interventions became the gold standard compared with the open surgical approach in both the non-pregnant and the pregnant population by decreasing the length of hospitalization and the wound infection rate [14]. We consider laparoscopy to be the method of choice in the first trimester of pregnancy due to the reduced size of the uterus, thus avoiding intraoperative accidents such as uterine perforation. Limitations of the operative visual field due to the increased size of the uterus will most often lead to increased intra-abdominal pressure and implicitly to possible adverse effects. The need for a high intra-abdominal pressure could harm both the mother and child by affecting hemodynamics [27].

The choice of the initial pressure of 12 mmHg is sustained by the report of Georgios Michos et al. as well as Ball E et al. regarding the laparoscopy performed during pregnancy. The conclusion of the studies was that the recommended intra-abdominal pressure for surgery should be as low as 12 mmHg, and also this value is proven to have no effect on fetal viability [27,28]. On the one hand, there are studies that describe performing laparoscopy during pregnancy with a pressure of 8 mmHg, demonstrating the possibility of a wide range when it comes to intra-abdominal pressure variation [16]. In our case, the abdominal pressure of 12 mmHg generally had negative effects on the patient manifested by pain felt in the right shoulder, which prompted us to suggest a decrease to 10 mmHg, improving the patient’s symptoms and compliance. Further research is needed to find a correlation between anesthetic dose, injection site, and maximum intra-abdominal pressure allowed in case of laparoscopy.

Evidence-based medicine shows that end-tidal carbon dioxide (ETCO_2_) should be monitored by reference in any laparoscopic intervention because of the risk of hypercapnia and possible fetal acidosis following increased intra-abdominal CO_2_ [27]. For this, we performed a non-invasive intraoperative measurement of CO_2_. The values varied between 33 and 36. We consider the ETCO_2_ measurement during combined spinal–epidural anesthesia a healthy practice to prevent possible acute decompensation of the patient due to anesthesia, the positioning of the patient to perform the laparoscopic procedure, an increase in intra-abdominal pressure, and physiological changes during pregnancy.

Also, analyzing several guidelines, we identified that the Doppler ultrasound of fetal heartbeats is sufficient to determine if fetal viability is preserved post-intervention [28]. We performed this procedure both preoperatively and postoperatively, as well as 24 h after the operation as a safety margin.

Another concern for pregnant women is related to the use of pain killer drugs. Our approach consisted of mounting the epidural catheter intraoperatively, which we later used in postoperative analgesia with ropivacaine at a concentration of 0.2%. We are aware that most classes of pain relievers are not fully studied during pregnancy and that there may be adverse effects. The only analgesic drug that we consider safe at this moment is acetaminophen, which we used in our case. In support of our hypothesis, we bring evidence both from studies and from Drugs.com, where we can see acetaminophen classified as AU TGA pregnancy category A, meaning that paracetamol is widely used by pregnant patients and that no negative effects have been observed [29]. Of course, there are studies that have investigated the use of pain relievers during pregnancy. A cohort study by Zafeiri A. et al. [30] analyzed the potential adverse effects of paracetamol, aspirin, naproxen, ibuprofen, and diclofenac on the fetus. For paracetamol, the only direct correlation shown was a reduced risk of having a high-birth-weight fetus, but for aspirin, ibuprofen, and naproxen, statistical significance was shown in terms of the increased risk of preterm birth, stillbirth, and admission of the baby to the neonatal unit. In the case of diclofenac, a lower risk of adverse effects but an increased risk of neural tube defects and hypospadias was demonstrated. We additionally studied the effects of paracetamol in pregnancy due to its widespread use, and in 2022, Castro C.T. et al. [31] conducted a study where no statistical significance could be found between women who used paracetamol during pregnancy and those who did not regarding preterm birth, low birth weight, and being small for gestational age. Due to the fact that paracetamol and other medicines are over the counter drugs, we consider overdose and long exposure time as determining factors of fetal malformations or postpartum fetal damage. Moreover, external factors such as illness, alcohol consumption, tobacco consumption, genetic factors, and many others that are recognized as being able to affect pregnancy must be taken into account.

In 2021, a meta-analysis was conducted by Tom Bleeser and Marc Van de Velde et al. [4], who identified 65 preclinical studies and no clinical studies about the negative effects of general anesthesia on the fetus. Preclinical studies conducted on animals led to the conclusion that there is a phenomenon of neurotoxicity due to general anesthesia. These conclusions could not be observed in the clinical setting. The phenomenon of neurotoxicity observed in the meta-analysis refers to learning and memory capacity, as well as neuronal damage (cell apoptosis, impairment of synaptic development, decrease in neuronal density, reduction in neuronal proliferation) resulting from prenatal exposure to general anesthesia. The anesthetics targeted in these studies were sevoflurane (most frequently mentioned), ketamine, propofol, isoflurane, enflurane, nitrous oxide, halothane, and desflurane.

In order to support the hypothesis of neuraxial anesthesia in the case of the pregnant patient, we sustain our hypotheses with recent publications that highlight the usefulness of spinal–epidural or combined spinal–epidural anesthesia in the case of the pregnant patient undergoing laparoscopic intervention. In 2021, Major A.L. et al. [16] prepared a paper in which they talk about the successful removal of a giant cyst in a pregnant patient by the laparoscopic technique under spinal anesthesia. The pregnancy was not affected by the surgical intervention or the anesthetic procedure. The patient later gave birth to a healthy child. Giampaolino P. et al. [17] report on the successful use of regional anesthesia for a pregnant patient in her 11th week of pregnancy. The anesthetic technique used is not specified. The subsequent follow-up of the pregnancy proves that the patient was at that moment in the 23rd week with a healthy fetus. Another publication of Maiti G.D. et al. [18] describes a patient in the 9th week of pregnancy who underwent laparoscopic salpingectomy under spinal anesthesia for a tubal ectopic pregnancy that ruptured, being in the situation of heterotopic pregnancy. The patient gave birth by cesarean section at 37th weeks to a healthy girl, with a normal recovery afterwards. Also, Wonte M.M. et al. [32] describes successful laparotomy in a pregnant patient under thoracic epidural anesthesia, thus opening up new doors regarding the approach to the pregnant patient. Of course, several publications were reported in the past in which regional anesthesia in the context of laparoscopy was a success, but we wanted to bring up-to-date relevant reports for these times. We consider our case a further reason to believe that combined spinal and epidural anesthesia can represent a feasible modality for the pregnant patient undergoing laparoscopic intervention.

We observe more and more the intervention of artificial intelligence (AI) on everyday life; thus, at some point, we are sure that AI will also enter the medical field. The present case is one under continuous research, so we expect frequent publications in upcoming years. Of course, physicians should be up to date with the new information, but they can never compare with a computer that can analyze hundreds of studies in a short period of time and even come up with a solution after comparing different results. In this sense, a publication about artificial intelligence in the diagnosis of sepsis/septic shock has been drafted and demonstrates how AI can diagnose signs and symptoms specific to sepsis faster than a human [33].

## 4. Conclusions

A patient in the 11th week of pregnancy who was initially treated with drugs that, according to most studies, lead to complications requiring emergency surgery due to both maternal and fetal risks. Pregnant patients, with specific physiological changes during pregnancy, are susceptible to the development of gallstones and therefore to complications such as acute cholecystitis and its potentially unfavorable evolution. Through our paper, we demonstrate that laparoscopic cholecystectomy can be a feasible approach in the case of pregnant women in the first trimester of pregnancy due to the many advantages compared to open cholecystectomy. To minimize any fetal risk, both intraoperatively and postoperatively, combined spinal and epidural anesthesia (CSEA) was the preferred technique, with the epidural catheter proving useful in postoperative analgesia using ropivacaine 0.2% (reduced systemic toxicity), limiting the number of potentially harmful drugs and providing patient satisfaction. We believe that this approach could represent a gold standard in the future. ETCO_2_ monitoring could be a good medical practice in pregnant women undergoing laparoscopic intervention because of the changes in ventilatory mechanics that may occur during the intervention as a result of increased intra-abdominal pressure and positioning of the operating table.

## Data Availability

No new data were created or analyzed in this study. Data sharing is not applicable to this article.
